# Supplementary material for: HIVprotI: an integrated web based platform for prediction and design of HIV proteins inhibitors
Source: J Cheminform. 2018 Mar 9;10:12. doi: 10.1186/s13321-018-0266-y (PMC5845081; doi:10.1186/s13321-018-0266-y)
Supplement: Supplementary file 2 — Additional file 2. Source code of HIVProtI web server. [file 13321_2018_266_MOESM2_ESM.zip › HIVprotI_Source-code/hivprotI/weka-3-6-6/doc/weka/core/matrix/LUDecomposition.html]

LUDecomposition


---


|  |  |  |
| --- | --- | --- |
| **Overview** \| **Package** \| **Class** \| **Tree** \| **Deprecated** \| **Index** \| **Help** \| **Weka's home** | |  |
| **PREV CLASS**   **NEXT CLASS** | **FRAMES**    **NO FRAMES**     **All Classes** |  |
| SUMMARY: NESTED \| FIELD \| CONSTR \| METHOD | DETAIL: FIELD \| CONSTR \| METHOD |  |


---


## weka.core.matrix Class LUDecomposition

```
java.lang.Object
  weka.core.matrix.LUDecomposition
```

**All Implemented Interfaces:**: java.io.Serializable, RevisionHandler

---

``` public class LUDecomposition extends java.lang.Object implements java.io.Serializable, RevisionHandler ```

LU Decomposition.

For an m-by-n matrix A with m >= n, the LU decomposition is an m-by-n
unit lower triangular matrix L, an n-by-n upper triangular matrix U, and a
permutation vector piv of length m so that A(piv,:) = L\*U. If m < n,
then L is m-by-m and U is m-by-n.

The LU decompostion with pivoting always exists, even if the matrix is
singular, so the constructor will never fail. The primary use of the LU
decomposition is in the solution of square systems of simultaneous linear
equations. This will fail if isNonsingular() returns false.

Adapted from the JAMA package.

**Version:**
:   $Revision: 1.4 $

**Author:**
:   The Mathworks and NIST, Fracpete (fracpete at waikato dot ac dot nz)

**See Also:**: Serialized Form

---

**Constructor Summary**

`LUDecomposition(Matrix A)`
  
          LU Decomposition


| **Method Summary** | |
| --- | --- |
| `double` | `det()`             Determinant |
| `double[]` | `getDoublePivot()`             Return pivot permutation vector as a one-dimensional double array |
| `Matrix` | `getL()`             Return lower triangular factor |
| `int[]` | `getPivot()`             Return pivot permutation vector |
| `java.lang.String` | `getRevision()`             Returns the revision string. |
| `Matrix` | `getU()`             Return upper triangular factor |
| `boolean` | `isNonsingular()`             Is the matrix nonsingular? |
| `Matrix` | `solve(Matrix B)`             Solve A\*X = B |

**Methods inherited from class java.lang.Object**

`equals, getClass, hashCode, notify, notifyAll, toString, wait, wait, wait`

**Constructor Detail**

### LUDecomposition

```
public LUDecomposition(Matrix A)
```

:   LU Decomposition

    **Parameters:**: `A` - Rectangular matrix


**Method Detail**

### isNonsingular

```
public boolean isNonsingular()
```

:   Is the matrix nonsingular?

    :   **Returns:**: true if U, and hence A, is nonsingular.

---


### getL

```
public Matrix getL()
```

:   Return lower triangular factor

    :   **Returns:**: L

---


### getU

```
public Matrix getU()
```

:   Return upper triangular factor

    :   **Returns:**: U

---


### getPivot

```
public int[] getPivot()
```

:   Return pivot permutation vector

    :   **Returns:**: piv

---


### getDoublePivot

```
public double[] getDoublePivot()
```

:   Return pivot permutation vector as a one-dimensional double array

    :   **Returns:**: (double) piv

---


### det

```
public double det()
```

:   Determinant

    :   **Returns:**: det(A) **Throws:**: `java.lang.IllegalArgumentException` - Matrix must be square

---


### solve

```
public Matrix solve(Matrix B)
```

:   Solve A\*X = B

    :   **Parameters:**: `B` - A Matrix with as many rows as A and any number of columns. **Returns:**: X so that L\*U\*X = B(piv,:) **Throws:**: `java.lang.IllegalArgumentException` - Matrix row dimensions must agree.: `java.lang.RuntimeException` - Matrix is singular.

---


### getRevision

```
public java.lang.String getRevision()
```

:   Returns the revision string.

    :   **Specified by:**: `getRevision` in interface `RevisionHandler`
    :   **Returns:**: the revision


---


|  |  |  |
| --- | --- | --- |
| **Overview** \| **Package** \| **Class** \| **Tree** \| **Deprecated** \| **Index** \| **Help** \| **Weka's home** | |  |
| **PREV CLASS**   **NEXT CLASS** | **FRAMES**    **NO FRAMES**     **All Classes** |  |
| SUMMARY: NESTED \| FIELD \| CONSTR \| METHOD | DETAIL: FIELD \| CONSTR \| METHOD |  |


---
